# Heat Stress-Induced Disruption of Endothelial Barrier Function Is via PAR1 Signaling and Suppressed by Xuebijing Injection

**DOI:** 10.1371/journal.pone.0118057

**Published:** 2015-02-18

**Authors:** Qiulin Xu, Jingxian Liu, Zhenglian Wang, Xiaohua Guo, Gengbiao Zhou, Yanan Liu, Qiaobing Huang, Lei Su

**Affiliations:** 1 Department of ICU, General Hospital of Guangzhou Military Command, Key Laboratory of Tropical Zone Trauma Care and Tissue Repair of PLA, Guangzhou, China; 2 Postdoctoral Workstation, Huabo Bio-pharmaceutical Research Institute, Guangzhou, China; 3 Southern Medical University, Guangzhou, China; 4 Guangzhou University of Chinese Medicine, Guangzhou, China; 5 Department of Pathophysiology, Southern Medical University, Guangzhou, China; Shanghai University of Traditional Chinese Medicine, CHINA

## Abstract

Increased vascular permeability leading to acute lung injury (ALI) and acute respiratory distress syndrome (ARDS) is central to the pathogenesis of heatstroke. Protease-activated receptor 1 (PAR1), the receptor for thrombin, plays a key role in disruption of endothelial barrier function in response to extracellular stimuli. However, the role of PAR1 in heat stress-induced endothelial hyper-permeability is unknown. In this study, we measured PAR1 protein expression in heat-stressed human umbilical venous endothelial cells (HUVECs), investigated the influences of PAR1 on endothelial permeability, F-actin rearrangement, and moesin phosphorylation by inhibiting PAR1 with its siRNA, neutralizing antibody (anti-PAR1), specific inhibitor(RWJ56110), and Xuebijing injection (XBJ), a traditional Chinese medicine used for sepsis treatment, and evaluated the role of PAR1 in heatstroke-related ALI/ARDS in mice by suppressing PAR1 with RWJ56110, anti-PAR1and XBJ. We found that heat stress induced PAR1 protein expression 2h after heat stress in endothelial cells, caused the release of endothelial matrix metalloprotease 1, an activator of PAR1, after 60 or 120 min of heat stimulation, as well as promoted endothelial hyper-permeability and F-actin rearrangement, which were inhibited by suppressing PAR1 with RWJ56110, anti-PAR1 and siRNA. PAR1 mediated moesin phosphorylation, which caused F-actin rearrangement and disruption of endothelial barrier function. To corroborate findings from in vitro experiments, we found that RWJ56110 and the anti-PAR1 significantly decreased lung edema, pulmonary microvascular permeability, protein exudation, and leukocytes infiltrations in heatstroke mice. Additionally, XBJ was found to suppress PAR1-moesin signal pathway and confer protective effects on maintaining endothelial barrier function both in vitro and in vivo heat-stressed model, similar to those observed above with the inhibition of PAR1. These results suggest that PAR1 is a potential therapeutic target in heatstroke.

## Introduction

Despite several decades of researches in pharmacologic therapy, heatstroke remains a major clinical problem with high morbidity and mortality and has a high incidence of multiple organ dysfunction syndromes (MODS). Acute lung injury (ALI) and acute respiratory distress syndrome (ARDS) are the most common complications in heatstroke and closely associated with prognosis, with the features marked by increased vascular permeability, tissue edema and extravascular effusions[1]. Diffused endothelium injury and disruption of endothelial barrier function leading to vascular endothelial hyper-permeability are central to the pathogenesis of ALI/ARDS. It is important to clarify the mechanisms of endothelial hyper-permeability induced by heat stress, which will provide novel insights in pharmacologic treatment for heatstroke.

Protease-activated receptor 1 (PAR1), a G protein-coupled, trans-membrane receptor, was identified as the first high-affinity thrombin receptor more than 20 years ago. PAR1 is expressed on the surface of nearly all cell types on the blood vessel wall, including endothelium, smooth muscle cells, platelets, neutrophils, and macrophages. Activation of PAR1 via proteolytic cleavage of its extracellular N-terminus by serine poteinase thrombin promotes platelet activation, cell proliferation, vascular development, and angiogenesis [2,3]. Studies also reported that PAR1 activated by matrix metalloprotease 1 (MMP-1) and thrombin regulates endothelial barrier function in some situations[2,4,5]. However, it remains unclear whether PAR1 is involved in heast stress-induced endothelial hyper-permeability.

Xuebijing injection (XBJ) is a traditional Chinese medicine approved for treatment of systemic inflammatory diseases, such as sepsis, ALI/ARDS, and acute kidney injury, by China Food and Drug Administration (CFDA). XBJ consists of extracts from five Chinese herbals: Carthami Flos, Paeoniae Radix Rubra, Chuanxiong Rhizoma, Salviae miltiorrhizae, and Angelicae Sinensis Radix. Recent report identified some active ingredients in XBJ, including senkyunolide I (SKI), safflor yellow A (SYA), oxypaeoniflorin (OPF), and benzoylpaeoniflorin (BOPF), to inhibit the activity of NF-κB, a key factor in inflammatory responses. We previously reported that XBJ inhibited inflammatory responses, attenuated liver injury and improve survival rates in heatstroke rats[6]. Likely, XBJ is a potential effective medicine in treating heatstroke, but studies that address the role of XBJ in heatstroke are insufficient.

In this study, we aimed to investigate the role of PAR1 in heat stress-induced endothelial hyper-permeability and further elucidated the protective mechanisms of XBJ against heatstroke. We found that PAR1 protein expression were induced by heat stress in a temperature-dependent manner, and inhibition of PAR1 with its specific inhibitor, RWJ56110, neutralizing antibody (anti-PAR1), and siRNA inhibited moesin phosphorylation and F-actin rearrangement, and decreased endothelial permeability. In vivo data showed that RWJ56110 and neutralizing antibody for PAR1 treatment attenuated microvascular permeability, pulmonary edema, protein exudation, and leukocytes adhesion in heatstroke mice. Moreover, XBJ inhibited PAR1 signal and decreased endothelial permeability in vitro and in vivo experiments.

## Materials and Methods

### Materials

RWJ56110 was purchased from Tocris Bioscience (Bristol, UK, Cat. 2614). XBJ was obtained from Tianjin Chase Sun Pharmaceutical Co., Ltd (Tianjin, China) with the batch number of 1303031, and stored at room temperature. Rabbit anti-PAR1 polyclonal antibody (Cat. sc-5605), mouse anti-PAR1 monoclonal antibody, ATAP2 (Cat: sc-13503), goat anti-phospho-moesin polyclonal antibody (Cat. sc-12895), mouse anti-MMP-1 monoclonal antibody (Cat. sc-21731), and rabbit anti-β-actin polyclonal antibody (Cat. sc-130656) were from Santa Cruz Biotechnology (CA, USA). Rabbit anti-moesin monoclonal antibody was from Abcam (UK, Cat. ab52490). HRP-conjugated rabbit anti-goat immunoglobulin G and goat anti-rabbit immunoglobulin G (Cat. ZDR-5308, ZB-5301) were from ZSGB-BIO (Beijing, China). The synthetic PAR1 peptides conjugated to keyhole limpet hemocyanin were used to generate polyclonal antisera in rabbits. IgG was purified by protein-A affinity chromatography to generate PAR1 IgG used as PAR1 neutralizing antibody in this study. The specificity of the MMP-1a Ab was tested by blocking the positive staining with the PAR1 peptide epitope in endothelial cells. senkyunolide I (SKI), oxypaeoniflorin (OPF), and hydroxysafflor yellow A (HYA) were from Y-S Biotechnology (Shanghai, China).

### Cell culture

Human umbilical vein endothelial cells (HUVECs) were purchased from Sciencell, and HUVECs at 10 ~ 20 passage were used for experiments. Cells were cultured in DMEM/F12 supplemented with 10% (*v/v*) fetal bovine serum (FBS), 100 U/ml of penicillin, and 100 μg/ml of streptomycin (Invitrogen Life Technology, USA) at 37°C in a humidified atmosphere of 5% CO_2_ and 95% air. All cell culture plates were coated with gelatin for 1 h.

### siRNA transfection

PAR1 siRNA (5`-GAACCCUGCUCGAAGGCUACUATT-3`) and negative control siRNA (5`-UUCUCCGAACGUGUCACGUTT-3`) were purchased from GenePharma Co., Ltd (Shanghai, China). HUVECs were transfected with siRNA using Lipofectamine RNAi MAX reagent (Invitrogen Life Technology, USA). After 48h of culture, the cells were replated, cultured for additional 24h, and used for experiments.

### Transendothelial electrical resistance (TER)

TER of monolayer HUVECs was determined using STX2 electrode and EVOM2 meter (World Precision Instruments, Sarasota, FL, USA) as described previously[7]. HUVECs were plated at 0.5×10^5^/well in gelatin-coated, 6.5 mm Trans-well filters (0.4 mm pore size), cultured for 3~4 days until full confluence, and starved in DMEM/F12 containing 0.5% FBS for 12h. To investigate the influences of temperature on endothelial permeability, HUVECs were transfected with PAR1 siRNA, or incubated with RWJ56110 (5μM), anti-PAR1 (25μg/ml), SKI (0.5μM), OPF (5μM), HYA (5μM), or XBJ at indicated concentration, then subjected to heat stress at 37°C, 39°C, 41°C, or 43°C, respectively for 2h, followed by TER examination. To investigate the effects of PAR1 or XBJ on endothelial permeability, cells were stimulated with 20μM RWJ 56110 and the indicated concentrations of XBJ, followed by heat stress at 42°C for 2h. Mono-layer endothelial TER values were obtained as baseline values before heat stress insult and measured at the indicated time after heat stress. The mean value of TER was expressed in the common unit (Ω cm^2^) after subtraction of the value of a blank cell-free filter. The percentage of TER relative to baseline value was calculated by the formula:

TER% = (TER of experimental wells / baseline TER of experimental wells) * 100

Every test was done in triplicate and repeated at least three times.

### Immuno-fluorscence

F-actin was stained using rhodamine-phalloidin as previously described[8]. Cells were plated in gelatin-coated glass-bottom microwells (MatTek) and cultured to confluence. After the appropriate treatments, cells were fixed and permeated for 15 min at room temperature in PBS with 3.7% formaldehyde and 0.5% Triton X-100, followed by incubating with rhodamine-phalloidin (Sigma Aldrich, USA) at 1:100 diluted in PBS for 1 h. Cell nuclei were counterstained with 1 μmol/L DAPI. Fluorescence was captured under the constant contrast and brightness conditions by a laser confocal scanning microscope (Zeiss, Germany).

### Western-blot

Following each treatment, lung tissue or cultured endothelial cells were lysed in RIPA buffer (50 mM Tris, pH 7.4, 150 mM NaCl, 1% NP-40, 0.5% sodium deoxycholate, 0.1% SDS, 1mM EDTA/EGTA/sodium vanadate, 10 mM β-glycerophosphate, and protease inhibitors: 1 mM phenylmethanesulfonylfluoride and 10 μg/ml of leupeptin/TPCK/aprotinin each) for 30 min on ice. Total proteins were separated by 12% SDS-PAGE gel and then transferred to PVDF membrane (Millipore, USA). Blots were probed with antibodies as follows: anti-PAR1 antibody (1:1000), anti-phospho-moesin antibody (1:1000), anti-β-actin (1:2000), anti-MMP-1 (1:1000), and anti-moesin (1:2000). Appropriate HRP-conjugated immunoglobulin G (1:5000) was used for secondary incubations. Reactive bands were visualized using the enhanced chemiluminescence system (Millipore, USA), detected using KODAK Image Station IS2000R (KODAK, USA), and quantified with Image J software.

### Animals Treatments

This study was conducted in strict accordance with the Guide for the Care and Use of Laboratory Animals of the National Institutes of Health and with approval of the Animal Ethics Committee of Guangzhou Military General Hospital. All surgery was performed under sodium pentobarbital anesthesia, and all efforts were made to minimize suffering. C57BL/6J male mice, 10–12 weeks, were purchased from Animal Resource Center of Southern Medical University. Animals were housed at an ambient temperature of 23 ± 1°C with a 12 hours light/dark cycle and allowed ad libitum access to food and water. Mice were administered with RWJ56110 (25 μg/kg, intraperitoneally), control IgG (2mg/kg, subcutaneously), or PAR1 neutralizing antibody (2mg/kg), followed by heatstroke induction. Heatstroke was induced as reported[9]. Briefly, mice were placed in an artificial climate chamber with an environment temperature of 38 ± 0.5°C and a relative humidity of 60% ± 5%. Rectal temperature (Tc) was measured every 10 minutes by a thermometer. Immediately after Tc reached 42.7°C, animals were taken out of the chamber and allowed to recover at room temperature (24 ± 2°C). Sham-heated animals underwent the same induction procedure of heatstroke with an environment temperature of 24 ± 0.5°C.

### Analysis of BAL fluid

Bronchoalveolar lavage (BAL) was performed by injecting 3 × with 0.8 ml PBS into the lung and gently aspirating the fluid. The BAL sample were centrifuged (500 × *g*, 15 minutes, 4°C) to obtain the cell pellet, supernatant was centrifuged again (16500 g, 10 minutes, 4°C), and pure BAL fluid was used to measure total protein. Cytoslides were prepared and stained with Diff-Quick (Dade Diagnostics, Deerfield, IL), and cell counts were determined using a hemocytometer. Pure BAL fluid stored for subsequent analysis of protein. Total protein concentration in supernatants of BAL using Bradford assay (Bio-Rad, Hercules, CA).

### Pulmonary microvascular leakage

At indicated time point, mice were anaesthetized with an intraperitoneal injection of sodium pentobarbital (50 mg/kg body weight) and Evans blue (20mg/kg, Sigma -Aldrich) was injected via tail vein 90 minutes prior to sacrifice. Pulmonary circulation was flushed with 5 ml PBS, and right lung was exercised and rinsed before being snap frozen in liquid nitrogen. Frozen tissue was homogenized in PBS (1ml/100μg tissue), incubated with 2 volumes of formamide at 60°C for 16 h, and centrifuged at 7000 g for 30 minutes. Absorbance of the supernatant was determined spectrophotometrically at 620 nm. The extravasated evans blue concentration was calculated against a standard curve and was expressed as a microgram of Evans blue dye per gram lung.

### Lung Wet/Dry Weight Ratio

Mice were killed by exsanguination under anesthesia. The left lung was harvested after reperfusion, weighed, and then placed in a vacuum oven (at 54°C) for 18 h. The ratio of lung wet weight to dry weight was then calculated as an indicator of pulmonary edema.

### Histopathology

For histopathology, lung from 4 mice per group were collected into 10% neutral buffered formalin, routinely processed into paraffin blocks, sectioned at 5 μm, and stained with hematoxylin and eosin in an automated slide stainer (Jung AutoStainer XL, Leica Microsystems). Lungs were visualized prior to histological analysis.

### Statistical analysis

All data were expressed as mean ± SEM and statistically analyzed by one-way ANOVA with Tukey`s post-hoc test using SPSS 13.0. A *p* value less than 0.05 was considered significant.

## Results

### Heat stress increases endothelial permeability and induces F-actin rearrangement

**Fig 1:**
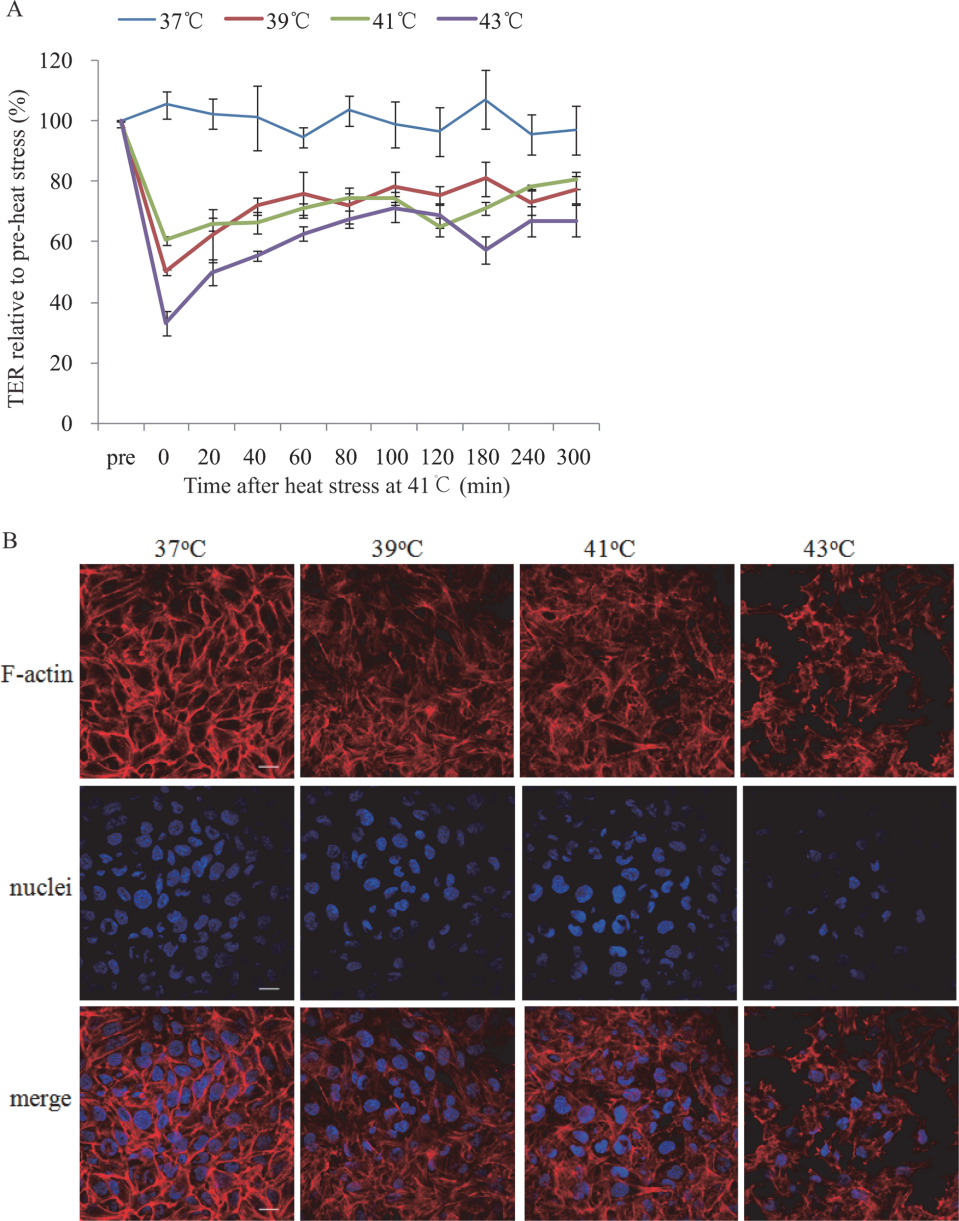
Influences of heat stress on Trans-endothelial resistance (TER) and F-actin rearrangement. **(a)** Mono-layer HUVECs were subjected to heat stress at 37°C, 39°C, 41°C, or 43°C for 2h, respectively, and TER was examined with an EVOM2 meter and a STX2 electrode at 37°C. Values are presented as a percentage relative to that prior to heat stress (n = 4, *P < 0.05, vs. 37°C, #P < 0.05, vs. 39°C). **(b)** Mono-layer HUVECs were subjected to heat stress at 37°C, 39°C, 41°C, or 43°C for 2h respectively, followed by recovery at 37°C for 2h, and then stained with DAPI (nuclei, blue) and rhodamine-phalloidin (F-actin, red). Representative F-actin, nuclei, and merged images were shown (scale bar: 20μm).

To test whether heat stress could increase endothelial permeability, monolayer HUVECs were subjected to heat stress at 37°C, 39°C, 41°C, or 43°C respectively for 2h, followed by repeated evaluation of TER at 37°C during 300 minutes post heat stress with an EVOM2 meter and a STX2 electrode. As shown in Fig. 1A, TER was decreased by heat stress and this effect sustained for over 300 minutes.

Actin in endothelial cells undergoes polymerization to form a double stranded filamentous structure known as F-actin, and is further assembled into three distinct cytoskeletal structures, including membrane skeleton, cortical actin rim and stress fibers. The balance between the centripetal tension generated by stress fiber and centrifugal tension by cortical actin rim regulate cell shape and inter-endothelial cell gap. Stress fiber formation in response to various environmental stresses will cause cell constriction and disruption of endothelial barrier function. In this study, cortical actin rim was complete and stress fiber was thin and obscure in cells at 37°C, while cortical actin rim disruption, inter-cell gaps widening, and stress fiber thickening and reorganization were observed in cells subjected to heat stress at 39°C, which became more significant in cells at 41°C and 43°C (Fig. 1B).

### Influences of heat stress on PAR1 protein expression

**Fig 2:**
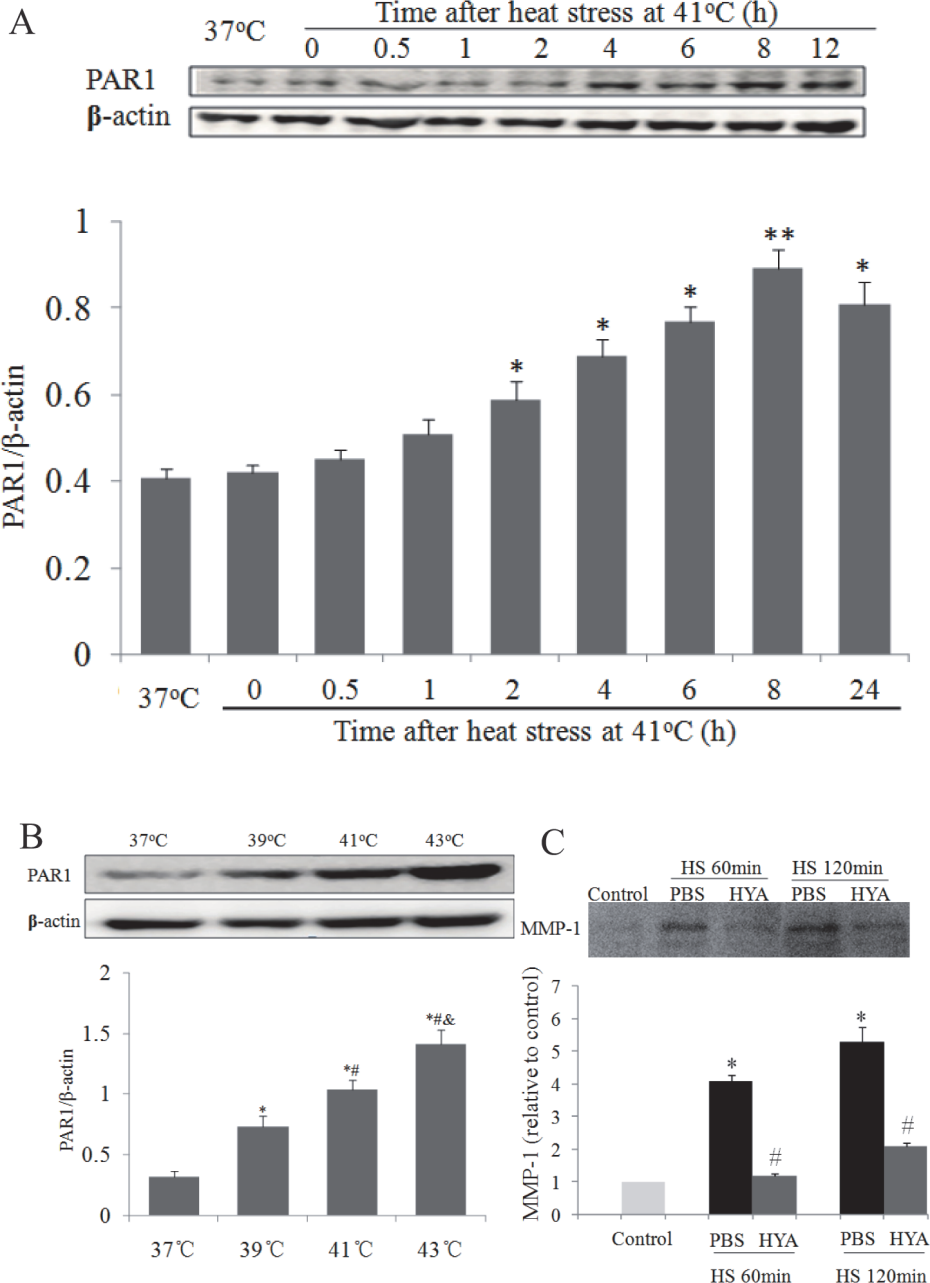
Influences of heat stress on PAR1 protein expression and releases of endothelial MMP-1. **(a)** Mono-layer HUVECs were cultured at 37°C or subjected to heat stress at 41°C for 2h, and time courses of PAR1 protein expression post heat stress were determined by western blot (n = 4, *P < 0.05, **P < 0.01, vs. 37°C). **(b)** Mono-layer HUVECs were subjected to heat stress at different temperatures for 2h, followed by recovery at 37°C for 4h, and PAR1 protein expressions were determined by western blot. Representative images of western blot and quantitative analysis of PAR1 protein normalized to β-actin were shown (n = 4, *vs. 37°C, ^#^vs. 39°C, ^&^vs. 41°C, P < 0.05). (c) Mono-layer HUVECs were subjected to heat stress (HS) at 41°C for 60 min or 120 min respectively, MMP-1 in medium were determined by western blot. Representative images of western blot and quantitative analysis of PAR1 protein normalized to β-actin were shown (n = 4, *vs. control, ^#^vs. PBS in corresponding HS group, P < 0.05).

PAR1 has been reported to be involved in increasing endothelial permeability in some situations, however, the role of PAR1 in heat stress-induced disruption of endothelial barrier function is not clarified. In this study, we first investigate whether heat stress influences PAR1 protein expression. Total proteins were extracted from endothelial cells immediately or 0.5h, 1h, 2h, 4h, 6h, 8h, and 24h after 2h of heat stimulation at 41°C. As shown in Fig. 2A, PAR1 protein expression was significantly increased at 2h, gradually reach a peak at 8h, and then slightly decrease from 8h to 24h after heat stress (Fig. 2A). The influences of heat stress temperature on PAR1 protein expression were also examined in endothelial cells 4h after heat stress at different temperature for 2h. PAR1 protein expression was significantly increased in the range of temperature from 39°C to 43°C in a temperature-dependent manner (Fig. 2B), which indicates that PAR1 is involved in heat stress-induced disruption of endothelial barrier function. Given that extracellular stimuli, such as thrombin or MMP-1, activated PAR1 in endothelial cells or epithelial cells, leading to the disruption of barrier function[4,5,10], we investigated whether heat stress induced releases of endothelial MMP-1 and thrombin. We found that MMP-1 in the medium was increased after 60 min or 120 min of heat stimulation at 41°C (Fig. 2C), while thrombin was not (data not shown).

### PAR1 mediated endothelial hyper-permeability and F-actin rearrangement induced by heat stress

**Fig 3:**
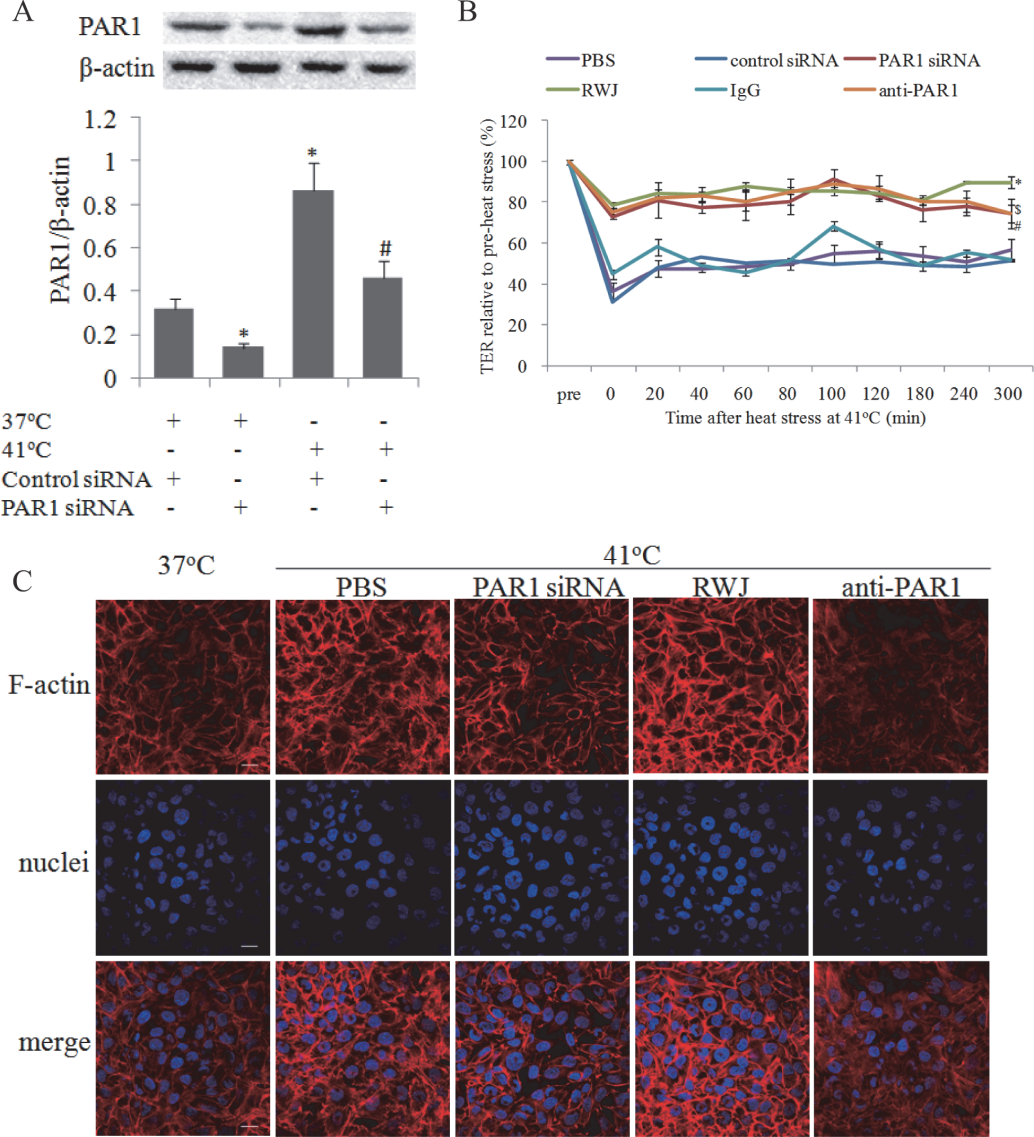
PAR1 mediated endothelial hyper-permeability and F-actin rearrangement induced by heat stress. Mono-layer HUVECs were treated with RWJ56110 (RWJ; 5μM), PAR1 neutralizing antibody (anti-PAR1), control IgG (IgG), PAR1 siRNA, or negative control siRNA, followed by heat stress at 41°C for 2h. Mono-layer HUVECs were transfected with control siRNA or PAR1 siRNA respectively, or treated with RWJ, anti-PAR1 and IgG, followed by heat stress at 41°C or cultured at 37°C for 2h, PAR1 protein expressions were determined by western blot. **(a)** Representative images of western blot and quantitative analysis of PAR1 protein normalized to β-actin were shown (n = 4, * vs. PBS group, P < 0.05). **(b)** TER was determined similar to Fig. 1A (n = 4, *P < 0.05, vs. PBS group, $ vs. control siRNA group, # vs. IgG group, P < 0.05). **(c)** Following above heat stress, the cells were recovered at 37°C for 2h, and nuclei (blue) and F-actin (red) were stained as Fig. 1B noted, scale bar: 20μm.

To examine whether PAR1 was involved in heat stress-induced elevation of endothelial permeability, we decreased PAR1 protein expressions in endothelial cells using siRNA transfection (Fig. 3A), or inhibited PAR1 activation with its specific inhibitor, RWJ56110, and its neutralizing antibody, ATAP2 (anti-PAR1). We found RWJ56110, anti-PAR1 and PAR1 siRNA attenuated heat stress-induced elevation of endothelial permeability, suggesting disruption of endothelial barrier function induced by heat stress is partially dependent on PAR1 (Fig. 3B, S1A).

The influences of PAR1 on F-actin rearrangement were evaluated by suppressing PAR1 with RWJ56110, anti-PAR1 and its siRNA prior to heat insults at 41°C. The characteristics of F-actin rearrangements, including cortical actin rim disruption, inter-cell gaps widening, and stress fiber thickening and reorganization, were abated by the inhibition of PAR1 activity and expression (Fig. 3C, S1B).

### Influences of PAR1 on the phosphorylation levels of moesin

**Fig 4:**
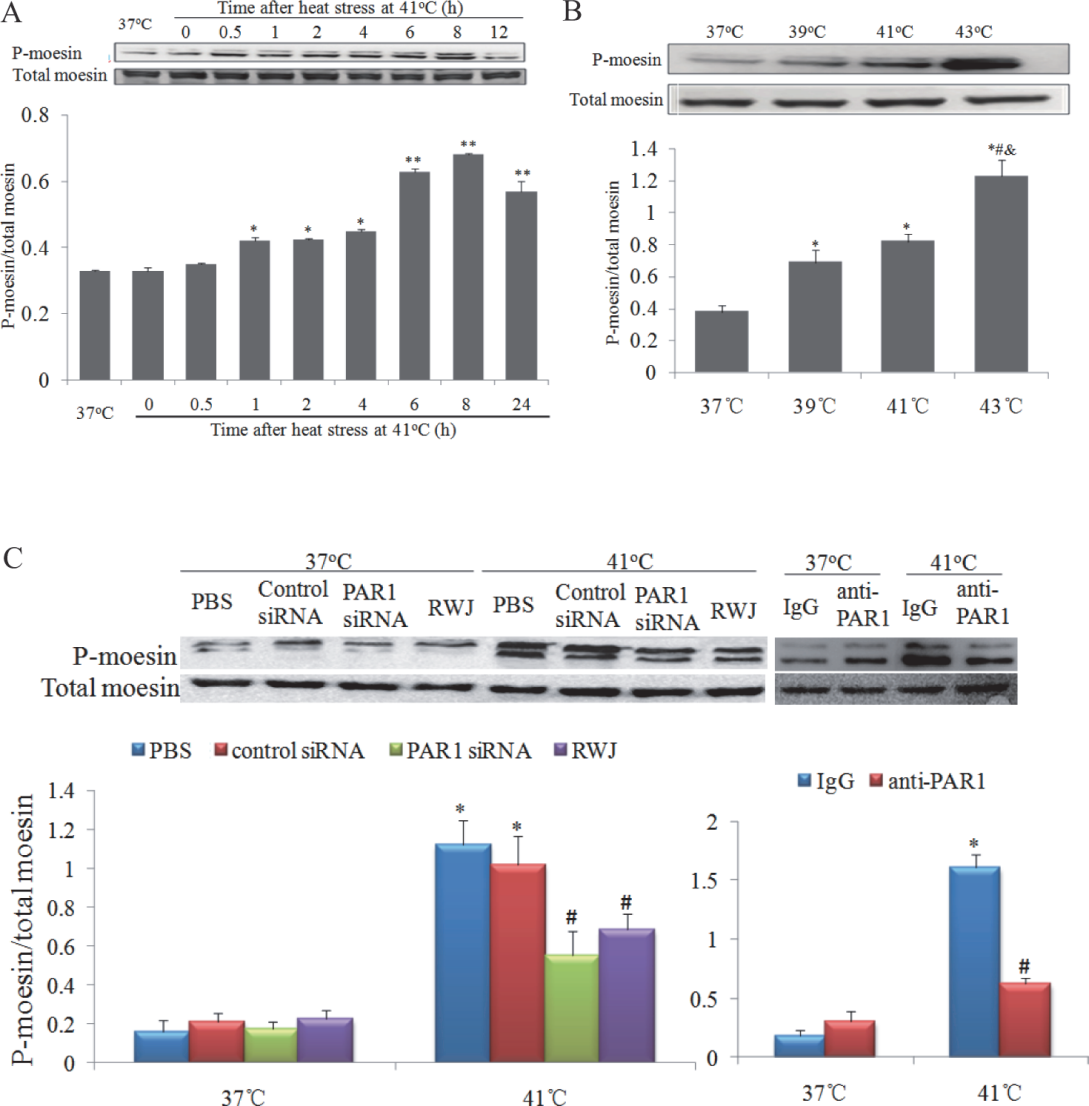
Heat stress increased phosphorylation levels for of moesin via PAR1 signal. Representative images of western blot and quantitative analysis of phosphorylation levels of moesin normalized to total moesin were shown (n = 4). **(a)** Mono-layer HUVECs were subjected to heat stress at 41°C for 2h, and time courses of moesin phosphorylation post heat stress were determined by western blot (*P < 0.05, **P < 0.01, vs. 37°C). **(b)** Mono-layer HUVECs were subjected to heat stress at different temperature for 2h, followed by recovery at 37°C for 2h, and phosphorylation levels of moesin was determined by western blot (*vs. 37°C, ^#^vs. 39°C, ^&^vs. 41°C, P < 0.05). **(c)** Mono-layer HUVECs were incubated with RWJ56110 (RWJ; 5μM), control IgG (IgG), anti-PAR1, PAR1 siRNA, or control siRNA, followed by heat stress at 39°C for 2h, and moesin phosphorylation was determined by western blot (*P < 0.05, vs. PBS at 37°C, ^#^P < 0.01, vs. corresponding control at 41°C).

We previously reported that moesin phosphorylation and F-actin rearrangement involved in advanced glycation end-products (AGEs) induced endothelial hyper-permeability [8,11,12]. In this study, we observed that the phosphorylation levels of moesin in endothelial cells were increased at 1h after heat stress, peaked at 8h, and slightly decreased at 24h after heat stress at 41°C (Fig. 4A). Moreover, the phosphorylation levels of moesin were further elevated as the temperature of heat stress increased from 39°C to 43°C (Fig. 4B). These results indicated that moesin phosphorylation was involved in heat stress-induced endothelial hyper-permeability. We further examined whether the levels of moesin phosphorylation were influenced by inhibiting PAR1 with RWJ56110 or its siRNA. We found RWJ 56110 or PAR1 siRNA significantly decreased the phosphorylation levels of moesin in endothelial cells subjected to heat stress at 41°C (Fig. 4C). Consistently, in vivo experiments showed that elevated PAR1 expression and moesin phosphorylation in in heatstroke mice were diminished by XBJ (S2 Fig.).

### XBJ inhibited PAR1-moesin signal pathway

**Fig 5:**
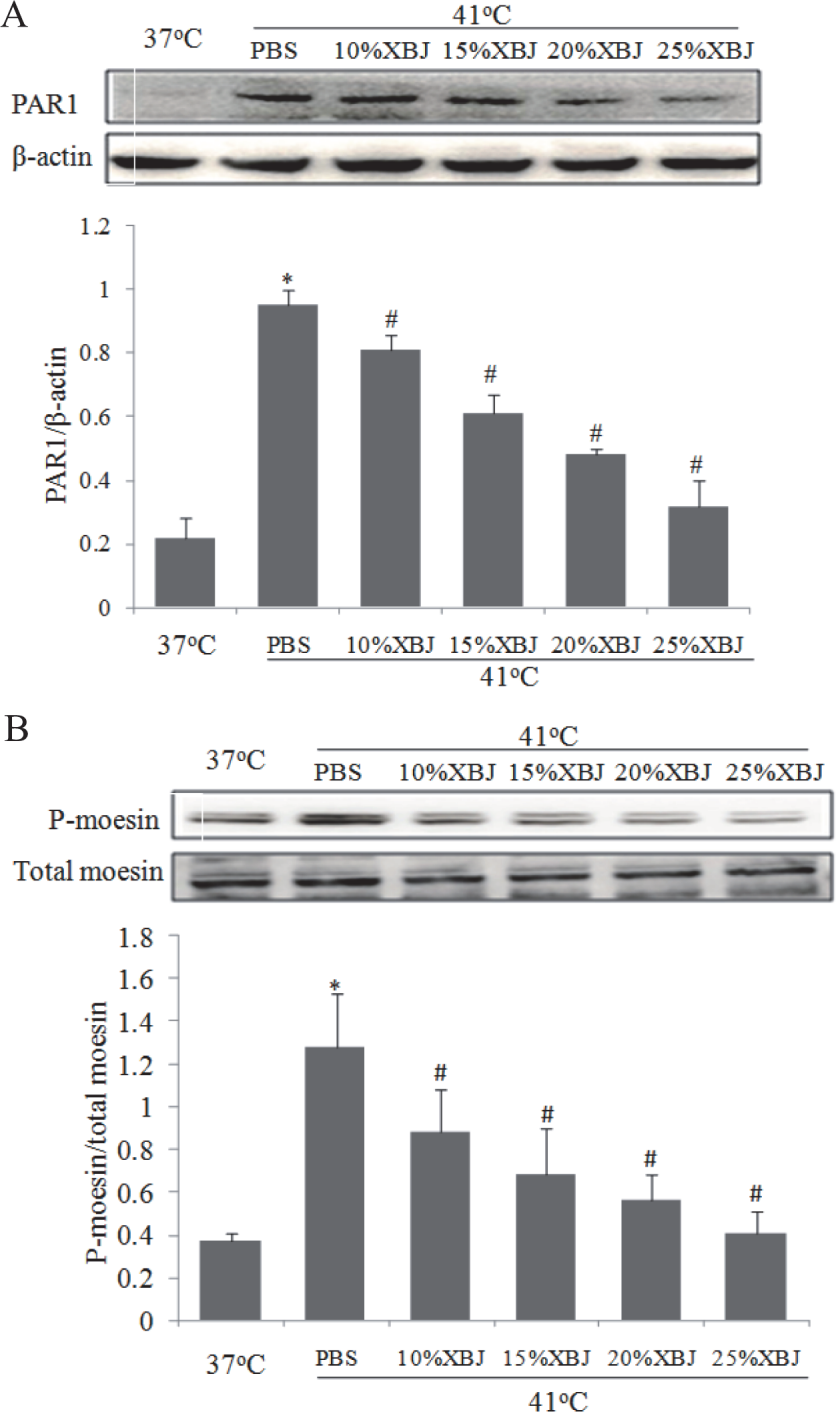
Influences of XBJ on PAR1 protein expression and phosphorylation levels of moesin. Mono-layer HUVECs were treated with PBS or XBJ at different concentrations for 30min, followed by heat stress for 2h at 41°C and recovery at 37°C for 2h. PAR1 protein **(a)** and phosphorylated moesin **(b)** were determined by western blot. Representative images of western blot and quantitative analysis of normalized protein levels were shown (n = 4, *P < 0.05, vs. 37°C, ^#^P < 0.05, vs. PBS at 41°C).

We have reported that XBJ protects rats from heatstroke-induced injuries and improves survival rates, while the underlying mechanisms remain unclear[6]. In this study, we found that heat stress-induced PAR1 protein expression and moesin phosphorylation were significantly decreased by XBJ dilutions in a concentration-dependent manner (Fig. 5A-B), indicating the protective role of XBJ is linked with the inhibition of PAR1-moesin signal pathway. However, we did not observed a further decrease in moesin phosphorylation in the cells treated with XBJ plus PAR1 siRNA (S3 Fig.).

### XBJ attenuated heat stress-induced endothelial hyper-permeability and F-actin rearrangement

**Fig 6:**
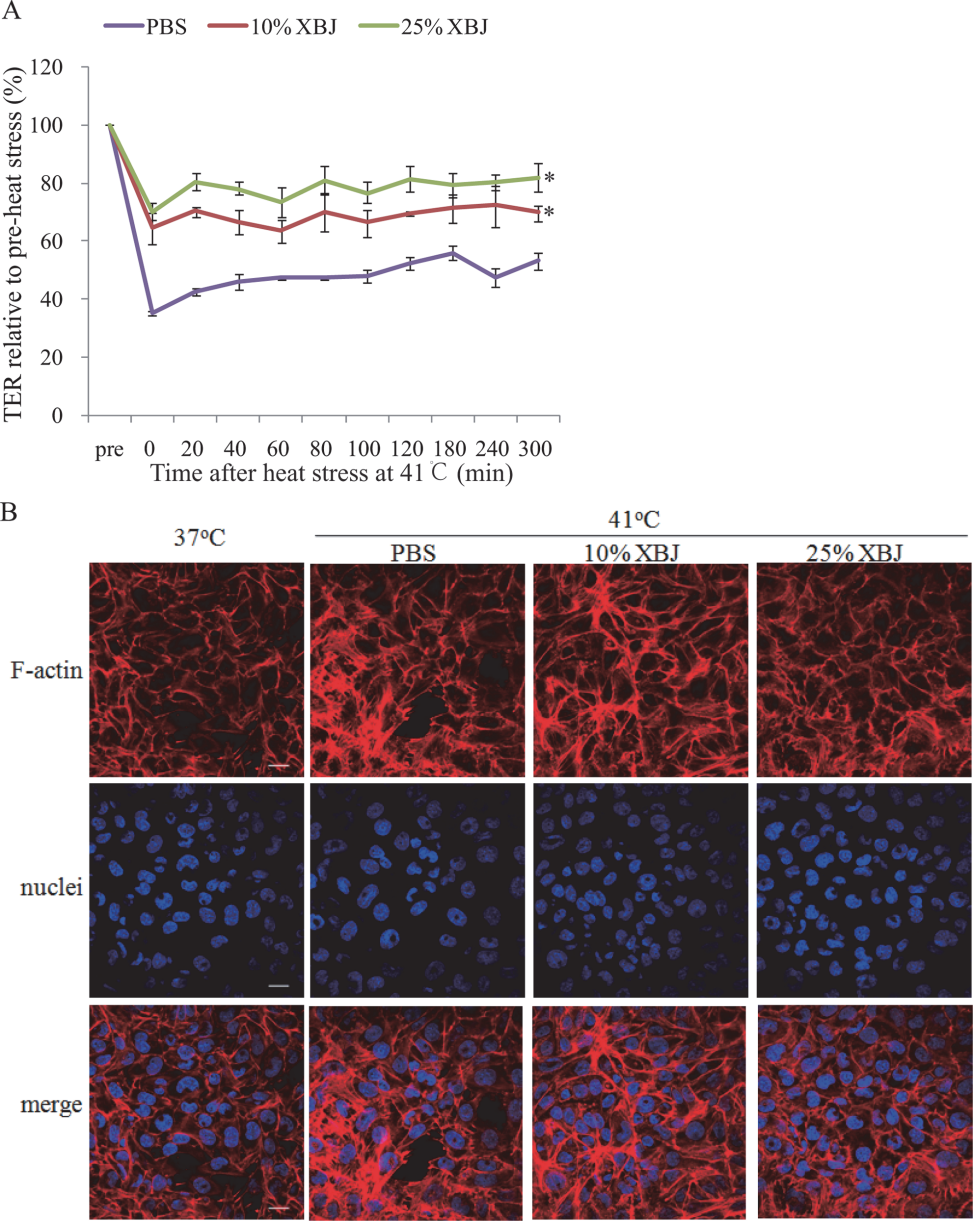
XBJ decreased endothelial hyper-permeability and F-actin rearrangement induced by heat stress. Mono-layer HUVECs were treated with 10% or 25% XBJ respectively for 30min, followed by heat stress at 41°C for 2h. **(a)** TER was determined similar to Fig. 1A (n = 4, *P < 0.05, vs. PBS). **(b)** Following above heat stress, the cells were recovered at 37°Cfor 2h, and nuclei (blue) and F-actin (red) were stained as Fig. 1B noted (scale bar: 20μm).

We next determined whether the inhibition of PAR1-moesin signal pathway by XBJ conferred a protective effect on endothelial barrier function and permeability in heat stress. The cells were pretreated with XBJ at a low (10%) or high concentration (25%) respectively used in above experiments, followed by heat stress at 41°C for 2h, and the influences of XBJ on endothelial permeability and F-actin rearrangement were evaluated. Both concentrations of XBJ inhibited heat stress-induced hyper-permeability and the changes of F-actin in endothelial cells (Fig. 6A-B), without such effects at 37°C (S1 Fig. A-B). We next investigated whether SKI, OPF, and HYA, the major active ingredients in XBJ, influenced endothelial permeability. We found that HYA decreased mono-layer endothelial TER induced by heat stress, while the other two ingredients did not (Fig. 4A,B).

### The influences of PAR1 and XBJ on pulmonary vascular permeability

**Fig 7:**
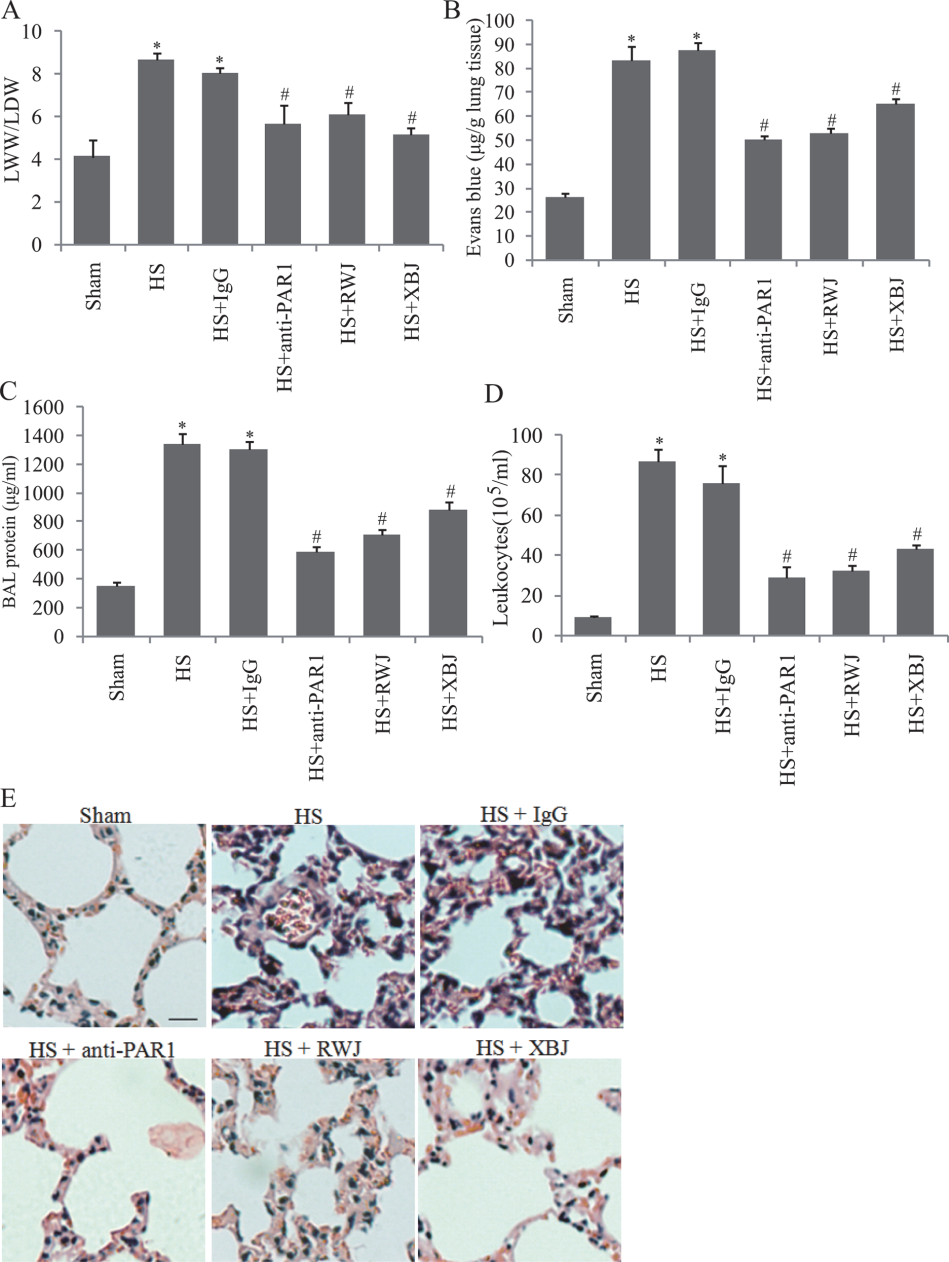
XBJ and suppression of PAR1 diminished ALI associated with heatstroke (HS). Mice were treated with XBJ (4ml/kg, intraperitoneally), RWJ56110 (RWJ; 25μg/kg, intraperitoneally), and PAR1 neutralizing antibody (anti-PAR1; 2mg/kg, subcutaneously) for 30min, followed by heat insult until Tc reached 42.7°C, and then recovered at room temperature for 2h. **(a)** Lung wet weight (LWW) to lung dry weight (LDW) ratio, **(b)** Envans blue leakage, **(c)** BAL proteins, and **(d)** BAL leukocytes numbers were determined (n = 6, *vs. sham, ^#^vs. HS, P < 0.05). **(e)** Representative images of lung stained with H&E (scale bar: 10μm).

The role of PAR1 and XBJ in the endothelial permeability in heat stress was tested in mice, a more complicated system than in vitro endothelial cells. Mice were pretreated with RWJ56110, neutralizing antibody for PAR1, and XBJ, followed by heat insult, and the pulmonary injury and microvascular permeability were evaluated. We found that increased LWW/LDW ratio, protein concentration and leukocytes in BAL, and Evans blue concentration in lung tissue were decreased by RWJ56110, neutralizing antibody for PAR1, and XBJ (Fig. 7A-D). Histological analysis demonstrated that Heat stress caused microvascular thrombi, neutrophils adhesion and pulmonary edema, which was also attenuated by PAR1 inhibitor, RWJ56110, neutralizing antibody, and XBJ (Fig. 7E). These data suggested that both inhibition of PAR1 and XBJ attenuated pulmonary microvascular permeability in heatstroke mice.

## Discussion

Respiratory failure is the most common complication in heatstroke with morbidity of about 85% and more than three quarters requiring mechanic ventilation as reported[13,14]. Increased vascular permeability leading to pulmonary edema is one of critical components in the pathogenesis of ALI/ARDS [15,16]. Although elevated vascular endothelial permeability induced by heat stress has been observed in mono-layer endothelial cells and animals [17,18,19], the mechanisms involved in this process are poorly understood. A major finding of this study suggests that PAR1 is involved in endothelial hyper-permeability in heat stress. Our data provide evidences that blocking PAR1 with the specific inhibitor, neutralizing antibody, or siRNA reduced endothelial hyper-permeability induced by heat stress, suggesting that PAR1 plays a role in the disruption of endothelial barrier function after exposure to heat stress. iIt is unclear whether and how PAR1 is activated by heat stress in cultured endothelial cells. Extracellular stimuli, such as thrombin or MMP-1, are reported to activate PAR1, causing vascular hyper-permeability [4,5,10]. In this study, we found that heat stress caused the releases of endothelial MMP-1, not thrombin, as early as 60 min after heat stimulation beginning, which may contribute to the activation of PAR1 signal. Likely, this activation seemed to happen in an autocrine manner, with endothelium as a major source of MMPs and thrombin [4,20]. Moreover, increased PAR1 expression in endothelial cells after heat stress stimulation appears to provide more substrates to be activated and support a much stronger signal transduction via activated PAR1, which contribute to the potent role of PAR1 in elevated endothelial permeability in heat stress.

In a more complicated in vivo system with heatstroke mice models, we found that blocking PAR1 with its specific inhibitor and neutralizing antibody diminished the disruption of pulmonary architecture integrity and tissue edema, proving the role of PAR1 in increased vascular endothelial permeability by heat stress. Interestingly, other characters of lung injury, including diffused thrombosis and inflammatory cell infiltration, were also ablated, suggesting that the promoting inflammation and coagulation function of PAR1 was also involved with ALI associated with heatstroke, which is correlated with the role PAR1 in sepsis reported by other literatures [4,21].

Important results in this study indicate that PAR1 is involved in regulation of moesin phosphorylation. Moesin has emerged to regulate endothelial permeability via modulating equilibrium between contractile forces generated by the endothelial cytoskeleton and adhesive forces produced at inter-endothelial junctions and cell-matrix attachment. We previously found that direct phosphorylation of moesin on threonine-558 by ROCK is required for the formation of stress fibers, the main contractile force in the endothelium, and the disruption of endothelial barrier function in response to AGEs stimulation[22]. The phosphorylation activation of Moesin is also observed in some other situations by Rho kinase, p38 MAPK, PKC, PI3K/AKT, and Rac1 [23,24,25]. Study reports that PAR1 activated by thrombin induces ERM phosphorylation, mostly moesin phosphorylation, in the endothelium, correlating with our data [5]. Collectively, other studies and our data suggest that PAR1 is a potential therapeutic target for ALI/ARDS associated with heatstroke.

Although numerous studies prove the clinical efficacy of XBJ and its anti-inflammation effects in sepsis, the role of in heatstroke is scarcely reported. Recently, Jun J, et al and we reported that XBJ diminished systemic inflammatory cytokines, attenuated liver injuries and improved survival in heatstroke rats [6,26], suggesting that XBJ is a potential therapeutic medicine for heatstroke. However, the role of XBJ in heatstroke is not fully clarified. In this study, we demonstrate that XBJ prevents heat stress-induced disruption of endothelial barrier function and vascular endothelial hyper-permeability both in vivo and in vitro, and that HYA, not SKI or OPF, contributes to such protective effects. We also provide evidences that XBJ decreases endothelial hyper-permeability via inhibiting PAR1 protein expression and moesin phosphorylation. Taken together, these results suggest the therapeutic role of XBJ in heatstroke through targeting PAR1 signal pathway.

In conclusion, vascular endothelial hyper-permeability plays a key role in ALI/ARDS associated with heatstroke. Our study suggests that PAR1 confers the detrimental effect on heat stress-induced disruption of endothelial barrier function and endothelial hyper-permeability, and is a potential pharmacologic target for heatstroke. Moreover, we demonstrate a new role of XBJ in maintaining endothelial barrier function via inhibiting PAR1-moesin pathway in heat stress.

## Supporting Information

S1 Fig RWJ, anti-PAR1, PAR1 siRNA, and 10% or 25% XBJ dilutions have no effects on TER and F-actin arrangement in endothelia cells sham-heated at 37°C. Mono-layer HUVECs were transfected with control siRNA or PAR1 siRNA respectively, or treated with RWJ, anti-PAR1 and IgG, followed by heat stress at 41°C or cultured at 37°C for 2h, PAR1 protein expressions were determined by western blot. (a) TER was determined as noted above. **(b)** Following above sham heat stress, nuclei (blue) and F-actin (red) were stained as noted above.
(EPS)

S2 Fig Influences of XBJ on PAR1 protein expression and phosphorylation levels of moesin in vivo. Mice were treated with XBJ (4ml/kg, intraperitoneally) or equal volume of PBS for 30min, followed by heat insult until Tc reached 42.7°C, and then recovered at room temperature for 2h. Lung tissues were harvested to extracted total protein. PAR1 protein and phosphorylated moesin were determined by western blot. Representative images of western blot and quantitative analysis of normalized protein levels were shown (n = 4, *P < 0.05, vs. 37°C, ^#^P < 0.05, vs. PBS at 41°C).
(EPS)

S3 Fig Influences of XBJ plus PAR1 siRNA on phosphorylation levels of moesin. Mono-layer HUVECs were transfected with PAR1 siRNA, or treated with XBJ, or XBJ plus PAR1 siRNA, followed by heat stress at 41°C or cultured at 37°C for 2h, phosphorylation levels of moesin were determined by western blot. Representative images of western blot and quantitative analysis of normalized protein levels were shown (n = 4, *P < 0.05, vs. 37°C, ^#^P < 0.05, vs. PBS at 41°C).
(EPS)

S4 Fig Influences of senkyunolide I (SKI), oxypaeoniflorin (OPF,), and hydroxysafflor yellow A (HYA) on mono-layer endothelial TER. (a) Mono-layer HUVECs were pretreated with SKI (0.5μM), OPF(5μM), or HYA(5μM), followed by incubation at 37°C **(a)** or heat stress at 41°C **(b)** for 2h, TER was determined as noted above(n = 4, *P < 0.05, vs. 37°C).
(EPS)
